# Bacterial diversity and community structure of salt pans from Goa, India

**DOI:** 10.3389/fmicb.2023.1230929

**Published:** 2023-12-04

**Authors:** Priti Gawas, Savita Kerkar

**Affiliations:** School of Biological Sciences and Biotechnology, Goa University, Taleigao, Goa, India

**Keywords:** salt pan, halophilic, Bacteria, diversity, next generation sequencing

## Abstract

In Goa, salt production from the local salt pans is an age-old practice. These salt pans harbor a rich diversity of halophilic microbes with immense biotechnological applications, as they tolerate extremely harsh conditions. Detecting the existence of these microbes by a metabarcoding approach could be a primary step to harness their potential. Three salt pans *viz.* Agarwado, Curca, and Nerul adjoining prominent estuaries of Goa were selected based on their unique geographical locations. The sediments of these salt pans were examined for their bacterial community and function by 16S rRNA amplicon-sequencing. These salt pans were hypersaline (400–450 PSU) and alkaline (pH 7.6–8.25), with 0.036–0.081 mg/L nitrite, 0.0031–0.016 mg/L nitrate, 6.66–15.81 mg/L sulfate, and 20.8–25.6 mg/L sulfide. The relative abundance revealed that the *Pseudomonadota* was dominant in salt pans of Nerul (13.9%), Curca (19.6%), and Agarwado (32.4%). The predominant genera in Nerul, Curca, and Agarwado salt pan sediments were *Rhodopirellula* (1.12%), *Sulfurivermis* (1.28%), and *Psychrobacter* (25.5%) respectively. The highest alpha diversity (Shannon-diversity Index) was observed in the Nerul salt pan (4.8) followed by Curca (4.3) and Agarwado (2.03). Beta diversity indicated the highest dissimilarity between Agarwado and the other two salt pans (0.73) *viz.* Nerul and Curca and the lowest dissimilarity was observed between Nerul and Curca salt pans (0.48). Additionally, in the Agarwado salt pan, 125 unique genera were detected, while in Nerul 119, and in Curca 28 distinct genera were noted. The presence of these exclusive microorganisms in a specific salt pan and its absence in the others indicate that the adjacent estuaries play a critical role in determining salt pan bacterial diversity. Further, the functional prediction of bacterial communities indicated the predominance of stress adaptation genes involved in osmotic balance, membrane modification, and DNA repair mechanisms. This is the first study to report the bacterial community structure and its functional genes in these three salt pans using Next-Generation Sequencing. The data generated could be used as a reference by other researchers across the world for bioprospecting these organisms for novel compounds having biotechnological and biomedical potential.

## Introduction

Salt production is an aged-old traditional method designed by farmers called *Mittkars* for over 150 decades. Salt pans are habitats inhabiting a huge community of extremophiles, thriving in extreme salinity, temperature, pH, and minimum nutrient availability (Mani et al., 2012). Halophilic bacteria from these environments have developed strategies and cellular systems that could cope with salt stress. Salt-in and salt-out are the two major strategies observed in halophiles to escape stress. In the salt-in strategy, the intracellular system is adapted to the salt concentration of the surrounding but the cellular energy consumed here is huge. Whereas, in salt-out strategies, organisms avoid adaptation and thereby energy loss by an accumulation of organic compatible solutes, also called a compatible solute strategy (Oren, 1999).

Halophilic bacteria from Goan salt pans are widely studied as a potent source of bioactive compounds (Tonima and Savita, 2011; Fernandes et al., 2019). The potential microorganisms in the salt pans of Goa have attracted the attention of the public as well as the researchers to conserve these habitats*, mittkars*, and their traditional knowledge of salt making. The culture-dependent approach examines a very minuscule microbial population. Modern methods such as high throughput sequencing and *in silico* analysis allow us to fill this gap (Janda and Abbott, 2007). The bacterial diversity of Agarwado, Curca, and Nerul salt pans and its comparative analysis has not been elucidated previously by next-generation sequencing. Hence, the present study is the first report accomplished to understand the diversity, distribution, and abundance with a comparative analysis of the bacterial community in these salt pans.

## Materials and methods

### Sample collection

**Figure 1:**
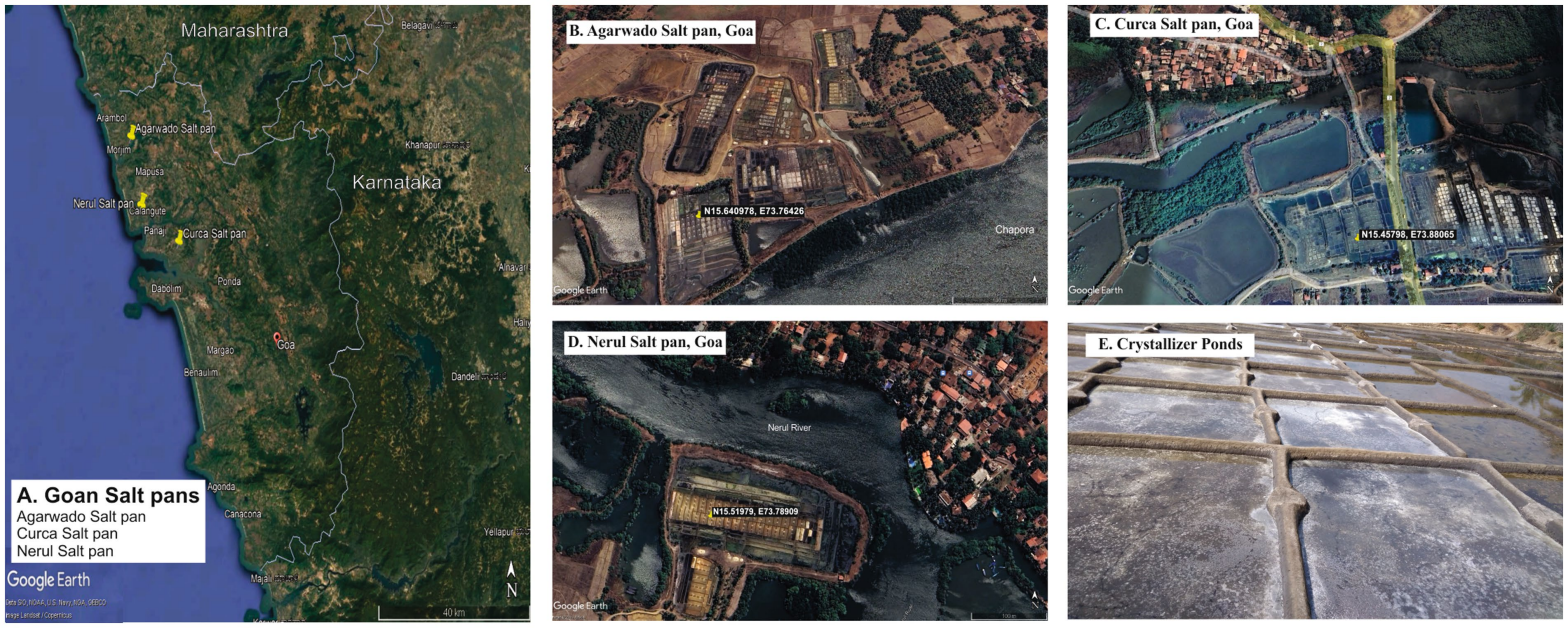
Goan Salt pans in study **(A)** Location of Agarwado, Curca, and Nerul Salt pans in Goa. Aerial image of **(B)** Agarwado salt pan **(C)** Curca salt pan **(D)** Nerul salt pan [Source: Google earth pro, 2023] **(E)** Image of crystallizer pond under solar evaporation at Agarwado salt pan.

Sediment samples were collected during the salt harvesting period from the crystallizer pond using a 5.5 cm diameter corer in sterile bags from the salt pans of Curca village (N15.45798, E73.88065) situated at Tiswadi taluka, Agarwado village (N15.640978, E73.76426) situated at Pernem taluka and Nerul village (N15.51979, E73.78909) situated at Bardez taluka (Figures 1A–E). Sediment sample refers to the sediments collected about 2 cm from a surface layer of salt pan. The sediment samples collected in triplicates from each salt pan site were mixed to obtain a single sediment sample. Sediment samples from Agarwado, Curca, and Nerul salt pans were indicated as AC, CC, and NC, respectively. Samples were transported in ice-cold condition to the laboratory and stored at −20°C until further processing. The temperature was recorded on-site using a thermometer of the three salt pan sediment samples. The pore water obtained after centrifugation of sediment samples was used to measure pH, salinity, nitrate, nitrite, sulfate, and sulfide. The pH was recorded by pH meter (Eutech, Germany) and the salinity was measured by refractometer (ATAGO, Japan). The nitrite and nitrate were estimated spectrophotometrically according to APHA (2012). Sulfate was estimated turbidometrically by measuring the precipitation of BaSO_4_ (Clesceri et al., 1998). For the estimation of sulfide, the sample was fixed in 2% zinc acetate and was further analysed spectrophotometrically (Cline, 1969).

### Environmental DNA extraction, library preparation, and Illumina sequencing

Environmental DNA was extracted from 1 g of each sediment sample using DNeasy PowerSoil Kit (Qiagen). The DNA obtained was checked for its concentration and quality using NanoDrop. The amplicon library was prepared using Nextera XT Index Kit (Illumina inc.) using bacterial specific forward primer 341F (GCCTACG GGNGGCWGCAG) and reverse primer 805R (ACTACHV GGGTATCTAATCC) for V3-V4 hypervariable region of 16S rRNA gene followed by adding Illumina adapters required for cluster generation. Amplicon libraries were purified by AMPureXP beads and quantified using a Qubit Fluorometer. Libraries were then loaded on MiSeq at 10–20 pM concentration for cluster generation and sequencing. The libraries were sequenced at Eurofins Genomics India Pvt. Ltd., Karnataka, India on the Illumina MiSeq platform using 2×300 bp chemistry to obtain paired-end reads.

### Sequence analysis by bioinformatics

Raw paired-end reads of AC, CC, and NC were analysed by Quantitative Insights Into Microbial Ecology 2 (QIIME 2), version 2–2023.7 (Bolyen et al., 2019). Reads obtained after sequencing were denoised via the q2-dada2 plugin (Callahan et al., 2016) which involves correction of Illumina sequenced errors, merging of reverse and forward paired-end reads, and clustering them into ASVs (amplicon sequence variants). The rarefaction curve was plotted using QIIME. ASVs obtained were further taxonomically classified into different taxonomic levels using the q2-feature-classifier plugin based on the SILVA (V.138) reference database (Quast et al., 2012). The Phylogenetic Investigation of Communities by Reconstruction of Unobserved States (PICRUST2) method (Douglas et al., 2020) was utilized to predict the functional potential of the bacterial communities in the three salt pans mentioned above. The relative abundance of functional protein profiles in the three salt pans was represented in a heatmap constructed using PRIMER7.

### Statistical analysis

The alpha (intra-sample) diversity indices [such as Simpson index, Shannon index, Menhinick’s richness index, and Pielou’s evenness (equitability)] were calculated at the genus level for the three sampling sites, similarly the beta diversity was measured as described by Whittaker (1960). Canonical Correspondence analysis (CCA) was done to understand the influence of environmental variables [Temperature (°C), pH, salinity (PSU), nitrate (mg/L), nitrite (mg/L), sulfate (mg/L), sulfide (mg/L)] on bacterial genera. Alpha diversity, beta diversity and CCA analysis were performed by using Paleontological statistics software (PAST), version 4.13 (Hammer et al., 2001).

## Results

### Sediment sample analysis

The physical parameters of sediment samples collected from Agarwado (AC), Curca (CC), and Nerul (NC) salt pans possessed temperatures of 35°C (in CC) and 36°C (AC and NC). Salinity observed was 450 PSU in AC and 400 PSU in CC and NC whereas the pH of the sediment samples was found to be 8.25 (AC), 7.6 (CC), and 8 (NC). The environmental DNA extracted from sediment samples AC, CC, and NC were measured by Nanodrop for its concentration and was found to be 28.6 ng/μl, 40.2 ng/μl, and 33.2 ng/μl, respectively.

### Relative abundance of bacterial community

**Figure 2:**
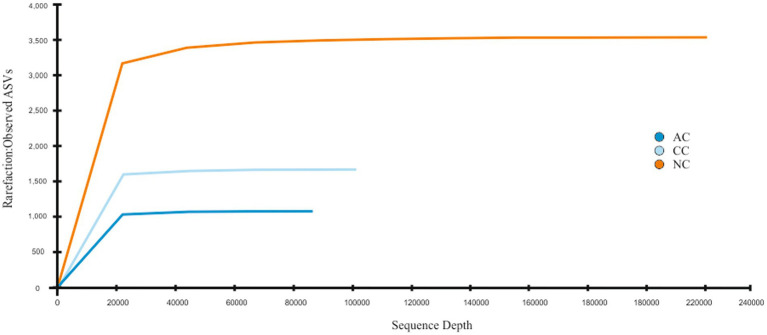
Rarefaction curve: Observed ASVs vs. Sequencing depth for three salt pan samples namely Agarwado salt pan (AC), Curca salt pan (CC), and Nerul salt pan (NC) of Goa, India.

A total of 95,299, 104,637, and 240,563 reads were obtained in sediment samples of the Agarwado (AC), Curca (CC), and Nerul (NC) salt pan, respectively. The rarefaction curve of all three samples reached a plateau at this sequencing depth (Figure 2) indicating the information contained in the three samples captured the majority of abundant phylotypes.

**Figure 3:**
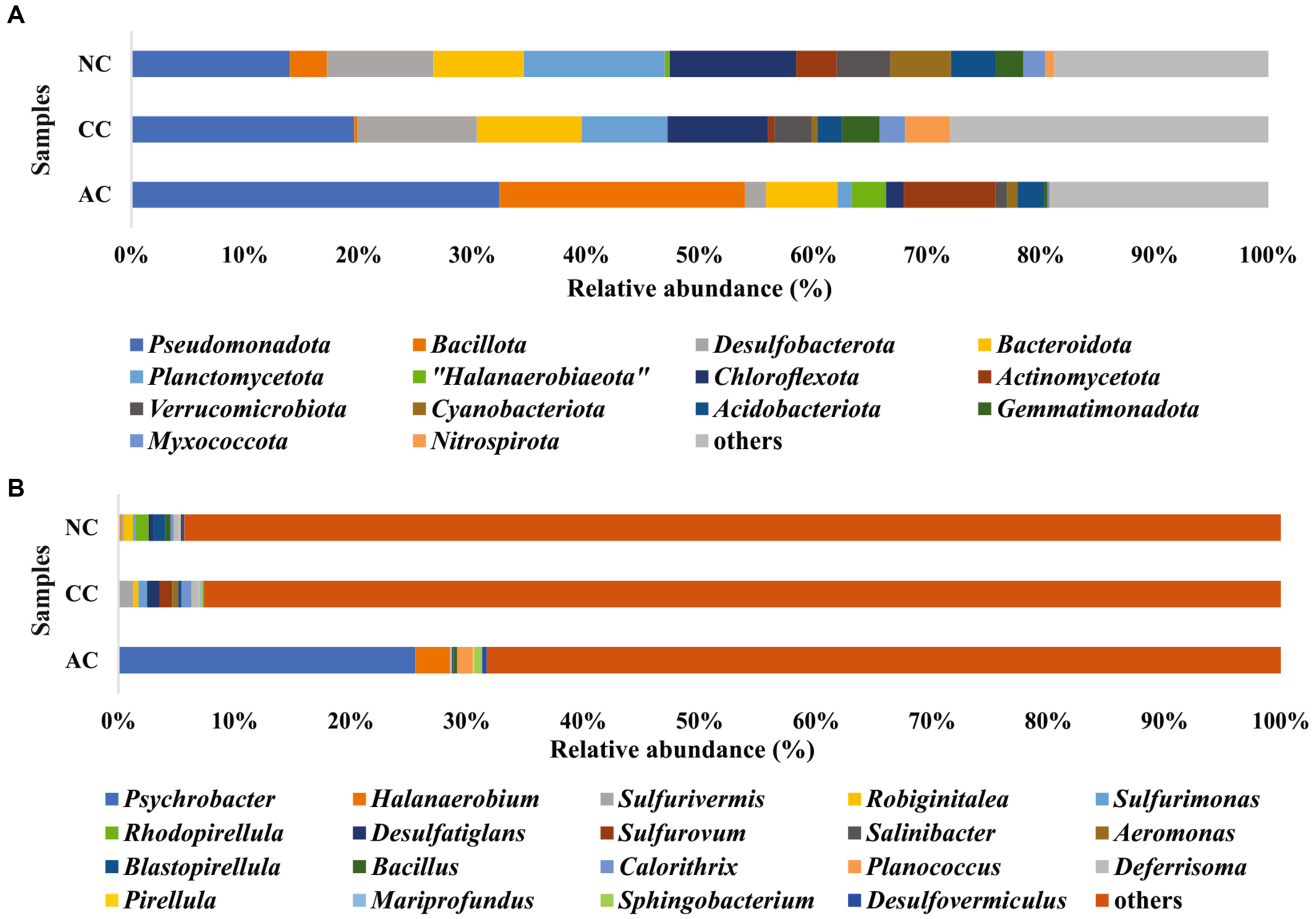
Relative abundance of bacterial communities at **(A)** phyla level and **(B)** genus level in sediment samples of Agarwado salt pan (AC), Curca salt pan (CC), and Nerul salt pan (NC) of Goa, India.

In the bacterial community analysis of the AC sample, 42 phyla and 201 genera were identified. The abundant phyla such as *Pseudomonadota* (33%), *Bacillota* (22%), and “*Candidatus* Patescibacteria” (9.4%), were observed (Figure 3A). Major contribution at the class level was by *Gammaproteobacteria* (30.7%), and *Bacilli* (20.7%). At the order level, *Pseudomonadales*, and *Bacillales* were found to be abundant with 26.8 and 19.4% relative abundance, respectively. Predominant taxa observed at the family level were *Moraxellaceae* (26.5%) and *Planococcaceae* (18.9%) and the major identified genera were *Psychrobacter* (26%), *Halanaerobium* (3.4%), and *Antarcticibacterium* (1.5%) (Figure 3B).

In the CC sample, 50 phyla and 143 genera were noted. The predominant phyla that contributed to bacterial diversity include *Pseudomonadota* (20.8%), *Desulfobacterota* (11.2%), and *Bacteroidota* (9.8%), (Figure 3A). At the class level, *Gammaproteobacteria* (17%) and *Bacteroidia* (8.4%) were found dominant. At the order level, the predominance of *Desulfobulbales* (5%) and *Desulfobacterales* (2.8%) were detected. At the family level, *Desulfobulbaceae* (3.9%) was relatively abundant. Lastly, at the general level, *Sulfurivermis* (1.3%) and *Desulfatiglans* (1.1%) presented a major proportion (Figure 3B).

In the NC sediment sample, 53 phyla and 250 genera were recognized. The dominant bacterial phyla include *Pseudomonadota* (14.7%), *Planctomycetota (*13%) and *Chloroflexota* (11.7%) (Figure 3A). The abundant classes observed were *Anaerolineae* (10.7%) and *Gammaproteobacteria* (9%). At the order level, *Pirellulales* with 6.5% relative abundance was found to be the highest followed by *Desulfobacterales* (3.8%). At the family level, *Pirellulaceae* (6.5%), was the major family obtained. Further, the most abundant genus identified was *Rhodopirellula* (1.2%) (Figure 3B).

### Correlation of bacterial genera with environmental parameters

**Figure 4:**
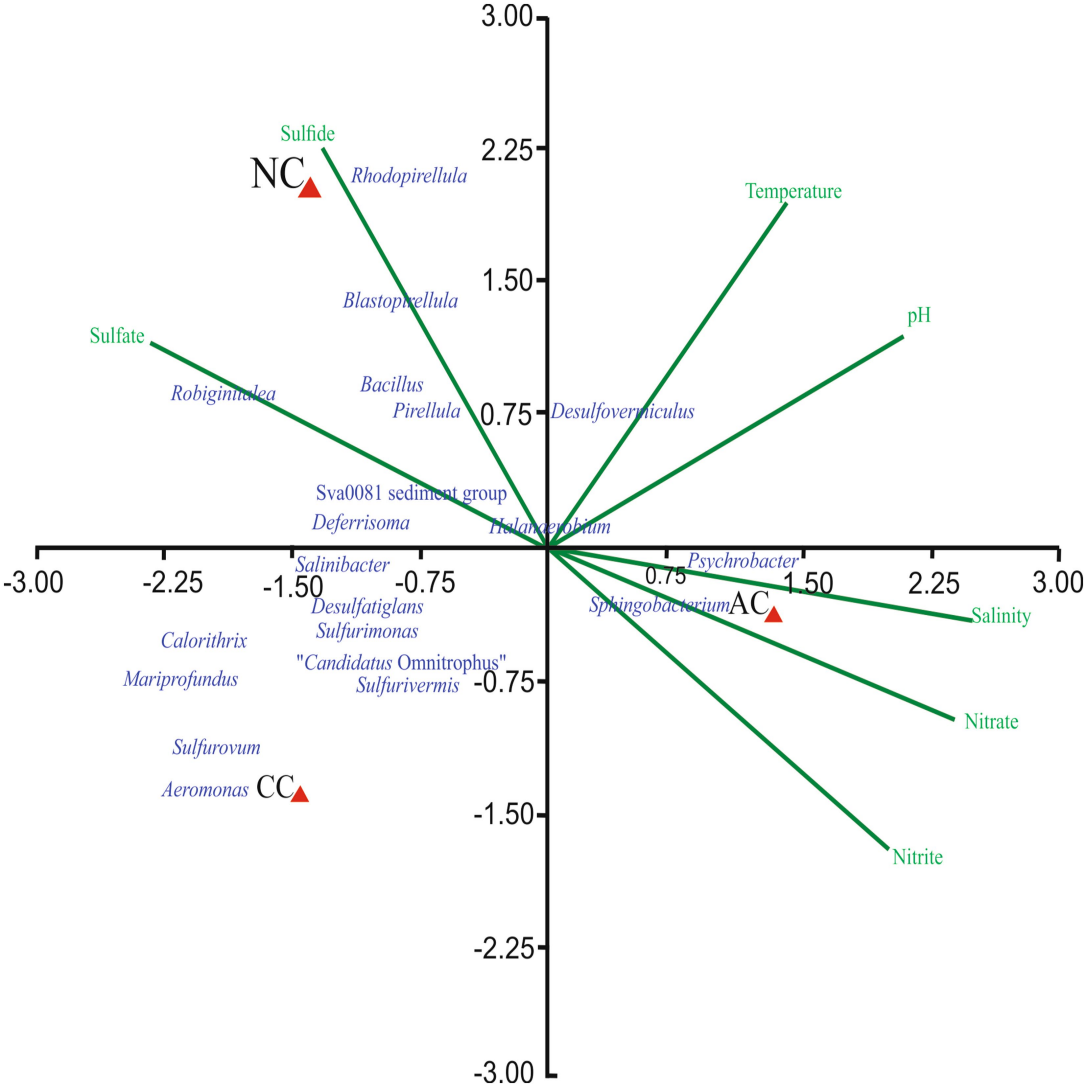
Canonical Correspondence Analysis ordination diagram of bacterial communities at generic-level at Agarwado salt pan (AC), Curca salt pan (CC), and Nerul salt pan (NC) of Goa, India associated with environmental parameters.

The effect of environmental parameters such as temperature, pH, salinity, nitrite, nitrate, sulfate, and sulfide were assessed on the bacterial genera using CCA. The CCA tripod explained 88.67% of the variation in bacterial genera by environmental parameters (Figure 4). The result revealed the positive correlation between genera *Blastopirellula*, Sva0081 sediment group, *Rhodopirellula*, *Robiginitalea*, *Pirellula* and *Bacillus* with sulfide and sulfate. However, genera *Psychrobacter, Halanaerobium*, *Planococcus* and *Sphingobacterium* found abundant in AC were positively correlated with nitrite, nitrate, and salinity. Of all the environmental parameters sulfide and sulfate contributed significantly to bacterial genera in NC.

### Alpha and beta diversity analysis at the genus level

The alpha diversity indices such as Simpson diversity index, Shannon diversity index (Shannon-Wiener index), Dominance, Menhinick’s richness index, and Pielou’s evenness (equitability) were calculated and depicted in Table 1. Similarly, the beta diversity and its pair-wise comparison presented in Table 2 were performed using the mathematical expression S/ā-1, where S is the total number of genera and ā is the average number of genera. The beta diversity was found highest and equal between AC and NC and AC and CC, i.e., 0.73. The lowest beta diversity was observed between CC and NC (0.48).

**Table 1:** Alpha diversity indices in Agarwado salt pan (AC), Curca salt pan (CC) and Nerul salt pan (NC) of Goa, India at genera level.

**Alpha diversity indices**	**AC**	**CC**	**NC**
Taxa (no. of genera)	201	143	250
Simpson diversity index	0.67	0.97	0.98
Pielou’s evenness (equitability)	0.38	0.87	0.86
Menhinick’s richness index	0.6	1	1
Dominance	0.32	0.02	0.01
Shannon diversity index	2.03	4.3	4.8

**Table 2:** Pair-wise comparison of beta diversity between Agarwado salt pan (AC), Curca salt pan (CC), and Nerul salt pan (NC) of Goa, India at the genus level measured by Whittaker using the mathematical expression S/ā-1, where S is the total number of genera and ā is the average number of genera.

	**AC**	**CC**	**NC**
**AC**	0	0.73	0.73
**CC**	0.73	0	0.48
**NC**	0.7	0.48	0

### Unique and common bacterial communities among the salt pans

**Figure 5:**
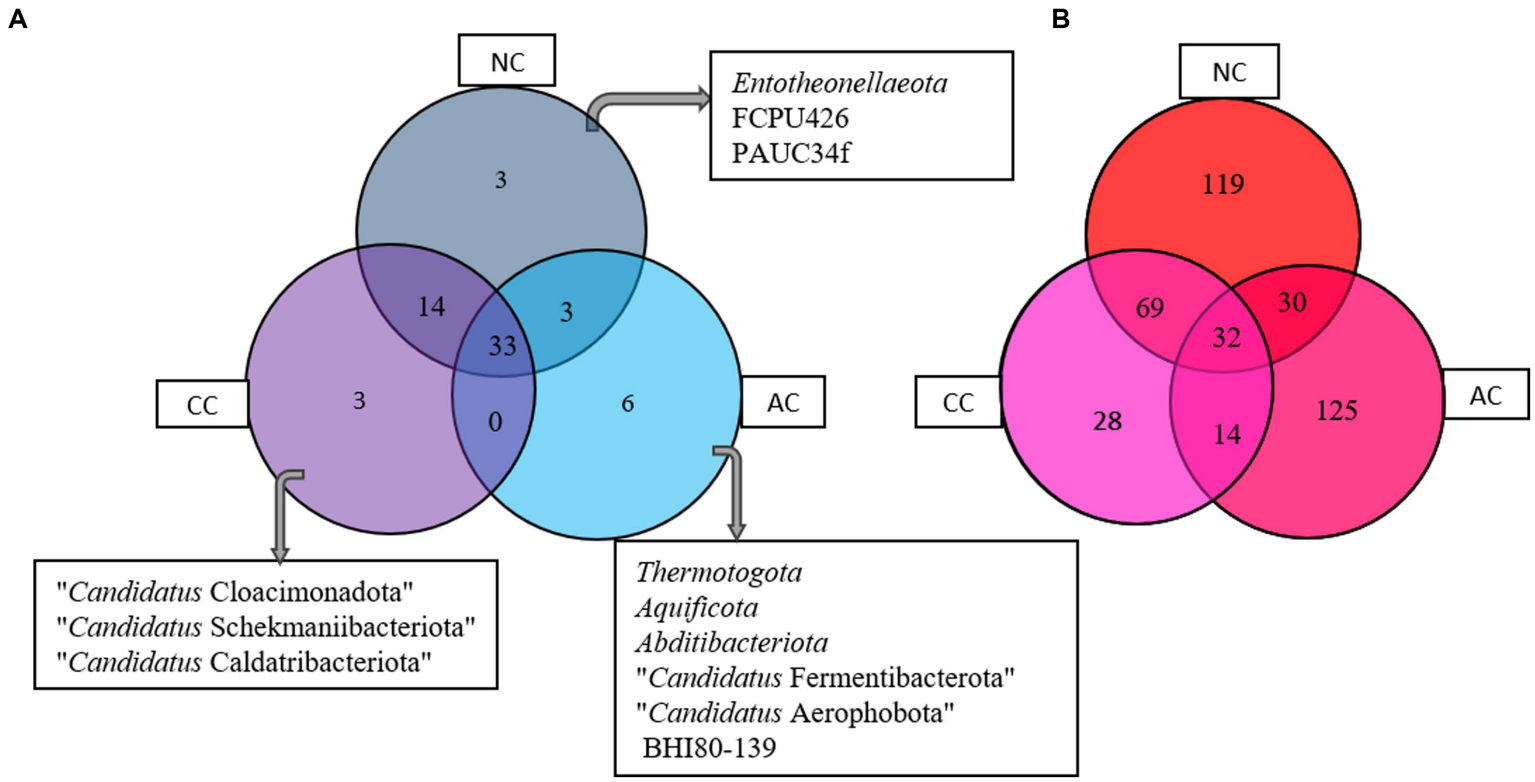
Venn diagram showing common and unique bacterial taxa at **(A)** phyla and **(B)** genus level between Agarwado salt pan (AC), Curca salt pan (CC), and Nerul salt pan (NC) of Goa, India.

The Venn diagram (Figure 5A) represents the number of unique and common phyla in three salt pans. An acceptably small percentage of unique taxon was observed in AC, CC, and NC with 14.2, 6, and 5.6%, respectively. Contrastingly, the common taxa observed in all three salt pans were in a higher proportion ranging from 62 -78%. There were no common phyla found in CC and AC. The phyla found common among all three salt pans are listed in the Supplementary Table S1.

At the genera level, a total of 201, 143, 250 genera were observed in AC, CC, and NC, respectively. Although the number of genera was found highest in NC, the percentage of exclusivity was observed high in AC (61%) > NC (47%) > CC (19.4%). The common genera found in all three salt pans ranged between 13 - 23%. The Venn diagram (Figure 5B) depicts the number of unique and common genera in the three salt pans and the genera are listed in the Supplementary Table S2.

### Rare taxa in the three salt pans

**Figure 6:**
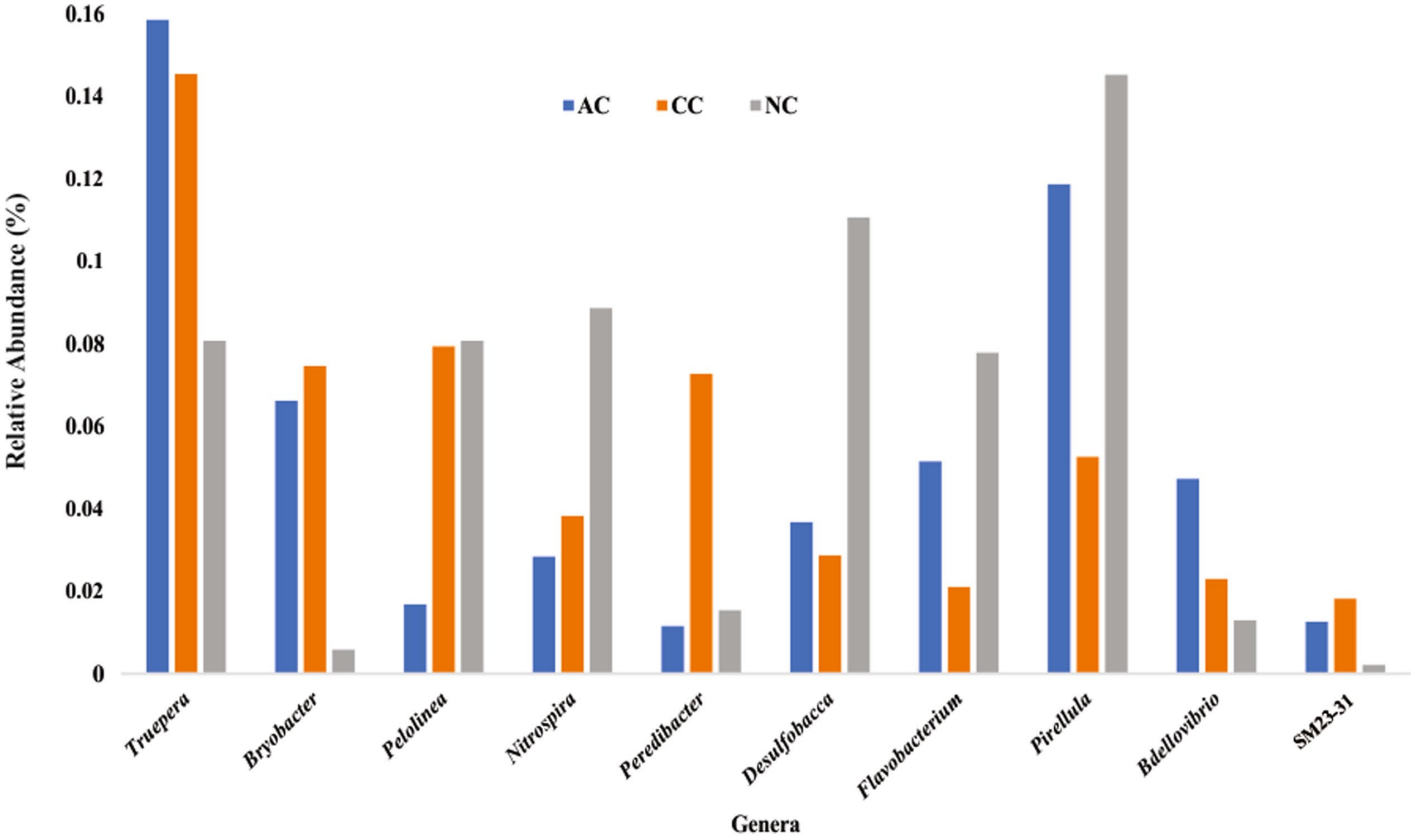
Rare bacterial genera (≤ 0.2% relative abundance) found common in all three sediment samples of Agarwado salt pan (AC), Curca salt pan (CC), and Nerul salt pan (NC) of Goa, India.

Rare taxa are defined as those having a relative abundance of ≤0.2% (Hugoni et al., 2013). At the phylum level, rare phyla such as *Bdellovibrionota* (0.06%), *Nitrospirota* (0.07%) and “*Candidatus* Sumerlaeota” (0.07%) were found in AC. In CC, “*Candidatus* Moduliflexota,” *Fusobacteriota,* and “*Candidatus* Acetithermota” are rare taxa with 0.04, 0.07, and 0.06% relative abundance, respectively. Further in NC, “*Candidatus* Babelota,” *Elusimicrobiota*, and *Deinococcota* are a few minor phyla with 0.11, 0.08, and 0.081% relative abundance, respectively. Secondly, “*Candidatus* Dadaibacteriota” was the only rare (≤ 0.2%) bacterial phyla found common in all three salt pans. Further, at the genus level only 11 taxa were detected common in all three salt pans (AC, CC, NC) with ≤0.2% of relative abundance (Figure 6).

### Functional gene prediction in three salt pans

**Figure 7:**
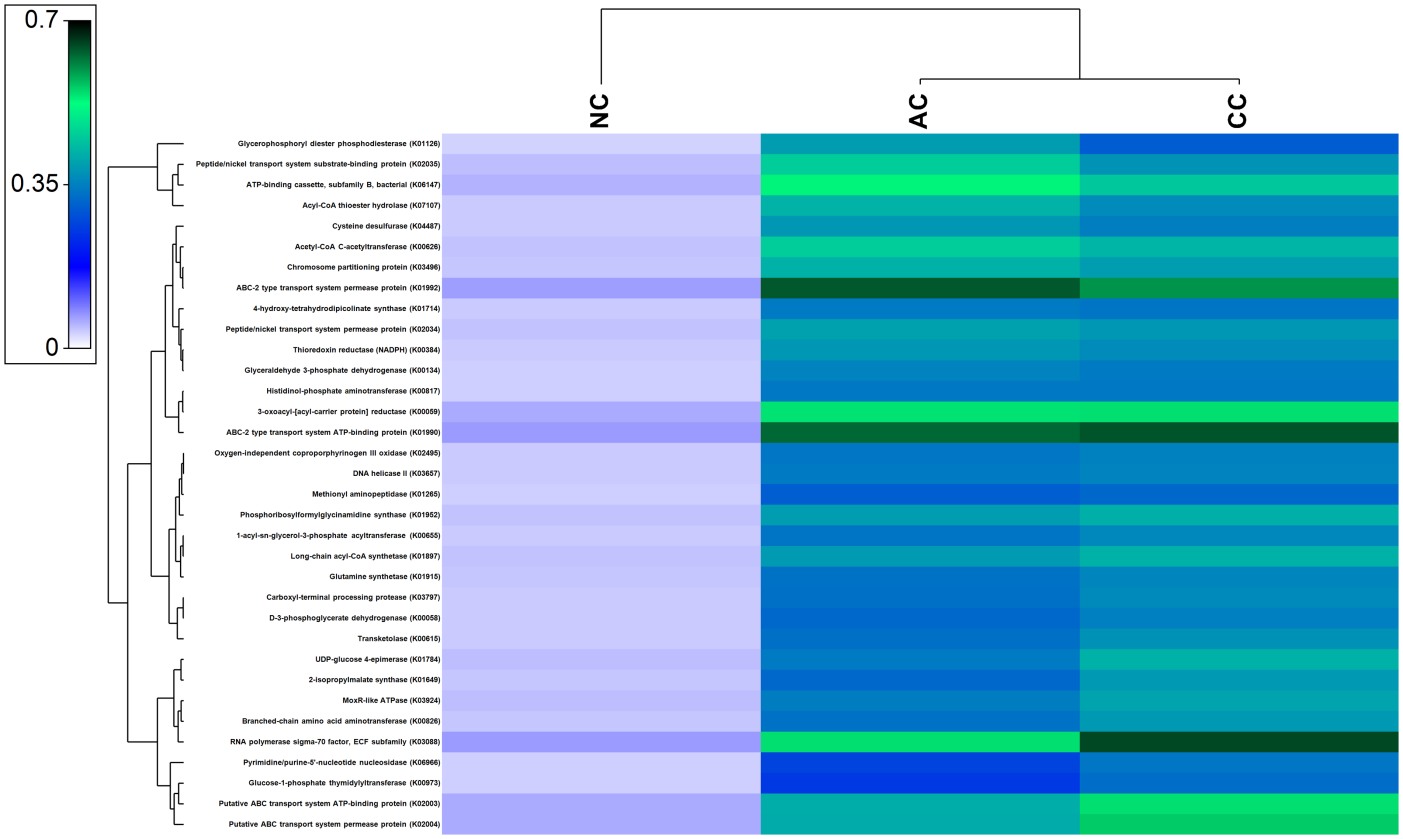
Heatmap showing the abundant functional genes predicted by using PICRUST2 algorithm in the sediment samples of Agarwado salt pan (AC), Curca salt pan (CC), and Nerul salt pan (NC) of Goa, India.

A large number of functional genes involved in various biological processes, including cellular metabolism and adaption to extreme environmental conditions, have been found in the indigenous bacterial communities of all the three salt pans. Figure 7 depicts the most abundant genes that were found in all the three salt pans. Further, numerous genes involved in the nitrogen, sulfur, and carbon cycles have also been found to be part of the overall functional gene profile (Supplementary Figures S1–S3).

## Discussion

Traditional salt-making is one of the major and unique occupations of Goa due to its lengthy coastline of 107 Km. The three study sites *viz.* Agarwado, Curca, and Nerul salt pans were selected based on their unique geographical locations. The Agarwado salt pan is in Pernem taluka adjoining the pristine Chapora estuary in the extreme north of Goa. The Nerul and Curca salt pans are connected to the two major lifeline estuaries of Goa, i.e., Mandovi in Bardez taluka and Zuari in Tiswadi taluka, respectively. Seawater from the estuary gushes into the salt pans through a sluice gate during high tide and is evaporated to produce salt. For decades, these three distinct salterns have been conventionally used to produce local salt by traditional methods in Goa. The temperature of 35°C - 36°C, noted from these salt pans during the salt harvesting period favors the evaporation process in the crystallizer pond to achieve the salinity required for salt production. The salinity of these salt pans was found to be in the range of 400–450 PSU and pH 7.6–8.25 indicating a halophilic and alkaline nature.

### Comparative analysis of the bacterial community in the salt pan

The bacterial composition of the salt pans of Goa revealed that the three sediment samples of salt pans were dominated by phylum *Pseudomonadota*. These results were parallel to the work reported by (Gorrasi et al., 2021) in other solar salterns. Within the *Pseudomonadota* phylum in the AC salt pan, only two classes such as *Gammaproteobacteria* and *Alphaproteobacteria* were observed. In addition to these two classes, *Zetaproteobacteria* was identified in CC and NC. Interestingly the only genera found in our study within the *Zetaproteobacteria* class was *Mariprofundus.* This class of bacterium is a model organism for microbial iron oxidation in marine environments (McAllister et al., 2019). The detection of genera *Mariprofundus* in CC and NC perhaps originated from the mining activities at Mandovi estuary which has deposited several tonnes of iron ore. Secondly, the Cumbarjua Canal interconnects the Mandovi and Zuari estuaries (adjacent to NC and CC respectively) and forms a network that has served as a major route for the transportation of iron ore. Additionally, the discharge of mining rejects from the mining sites has also concentrated iron particles in these estuaries (Gauns, 2011). This demonstrates how the distribution of microorganisms in a particular area is affected by their chemical composition. Similarly, other environmental parameters like salinity, nitrite, nitrate, sulfate, and sulfide also have an influence on bacterial distribution and abundance as depicted by CCA. The dominant phyla observed in our study such as *Pseudomonadota, Bacteriodota*, *Chloroflexota,* etc. are found to have ubiquitous genetic machinery and survival weapons (Mehta et al., 2021). Hence, understanding the microbiome composition, evolving genetic machinery, and adaptive mechanisms evolved by these extremophiles make it important in biotechnology industries. The genera *Psychrobacter*, belonging to the family *Moraxellaceae*, was the most abundant genera and family, respectively, in the AC with approximately 25.5% relative abundance. Like our studies, the presence of *Psychrobacter* was also reported in the solar saltern of Bohai Bay, China, revealed by DGGE of the 16S gene (Zhang et al., 2016). The isolation of sulfidogenic bacteria *Halanaerobium,* reported from the Ribandar salt pan of Goa was observed to be the second abundant genera in AC (Das et al., 2017). The abundant genera observed in the CC and NC salt pans were *Sulfurivermis* and *Rhodopirellula, respectively.* The presence of *Sulfurivermis* shows the occurrence of active sulfur cycles in the salt pan being a sulfur oxidizing bacterium (SOB) that uses nitrate as an electron acceptor in the rTCA pathway (Kojima et al., 2017).

### Alpha and Beta diversity analysis of the salt pans

The diversity of any ecosystem depends on two components “richness and evenness” (Soininen et al., 2012). In the Menhinicks richness index, an equal richness value was observed for NC and CC and was higher in comparison to AC, evident for higher community richness in Nerul and Curca salt pans. Similarly, the Pielou’s evenness index for NC and CC was found to be higher as compared to AC, which indicates the distribution of an equal number of taxa in both salt pan (NC and CC) ecosystems than in the Agarwado salt pan. Further, dominance in AC was higher than NC and CC, demonstrating the presence of a more dominant taxon in the Agarwado salt pan community. Lower evenness and higher dominance observed in AC indicate lower alpha diversity. However, in Nerul and Curca salt pans high evenness and lower dominance indicate high alpha diversity. A similar observation was shown by the Shannon diversity index which considers both the richness and evenness of a community. The alpha diversity measured by the Shannon index was NC > CC > AC.

Interestingly equal dissimilarity diversity (beta diversity) was observed between AC and NC and AC and CC, which further indicated that both CC and NC are quite comparable to each other but highly divergent to AC. The similarity between NC and CC was further confirmed by the presence of high number of common genera in both (represented in Figure 5B) in addition to the beta diversity. This is due the adjoining estuaries at Nerul and Curca salt pans that are connected by the Cumbarjua canal and therefore share uniform environmental characteristic (Qasim and Gupta, 1981) influencing bacterial composition.

### Bacterial taxa in ecological roles

*Pseudomonadota*, *Planctomycetota*, and *Chloroflexota* observed in the three salt pans are representatives of the commonly occurring phyla that are involved in a variety of ecological roles. The sulfur cycle advances in tandem with the carbon cycle and is most prevalent in hypersaline environments. Using sulfate as an electron acceptor, the sulfate-reducing bacteria break down organic molecules in an anoxic environment thereby cycling carbon anaerobically (Kerkar and Bharathi, 2007). In the current anticipated functional metagenome, genes involved in assimilatory and dissimilatory sulfate reduction and oxidation of sulfur compounds were found. The reductive nature of the salt pans sulfur cycle in the current work is indicated by the presence of the genera *Desulfovermiculus* and *Desulfatiglans* (Begmatov et al., 2021) in addition to genes such as adenylylsulfate reductase, subunits A (K00394), adenylylsulfate reductase, subunits B (K00395), dissimilatory sulfite reductase alpha subunit (K11180), dissimilatory sulfite reductase beta subunit (K11181), and sulfate adenylyltransferase (K00958). Likewise, different genes of the Sox system that are involved in sulfur oxidation: SoxA (K17222), SoxX (K17223), SoxB (K17224), SoxY (K17226), SoxZ (K17227), SoxC (K17225) and Sulfane dehydrogenase subunit were observed in the functional gene prediction (Supplementary Figure S3). *Thiohalospira* is a genus of halophilic, chemolithoautotrophic SOB that utilizes sulfur compounds as electron donors (Sorokin et al., 2020); it has only been found in the Agarwado (AC) salt pan. Secondly, the SOB genus, *Sulfuriflexus* (class: *Gammaproteobacteria*) (Kojima and Fukui, 2016; Rana et al., 2020), was exclusive to Curca (CC) sediments. *Sphingobacterium* and *Woeseia* functional in carbon-fixing (Lohmann et al., 2020) were also observed in these salt pans along with numerous genes required in carbon metabolism (Supplementary Figure S1). Unique to the Nerul (NC) salt pan, a group at genus level Cm1-21 (family: *Nitrosococcaceae*) was recently reported as ammonium oxidizers involved in the nitrification process of the nitrogen cycle (Semedo et al., 2021). Besides functional genes (Supplementary Figures S1–S3), bacterial genera like *Desulfovibrio* (observed in AC and NC sediment samples), *Pseudomonas* (AC and CC), *Nitrospina* (CC and NC), and *Nitrospira* (AC, CC, and NC) noted in our study are implicated in the carbon, nitrogen, and sulfur cycles (Megonigal et al., 2003; Meng et al., 2022; Sun et al., 2022), which illustrates that the movement of nutrients necessary to support the persistent life in these man-made salt pan ecosystems are governed by the presence of its indigenous microbes.

### Significance of halophiles

Salinity is a key factor that governs the microbial community in hypersaline ecosystems such as solar saltern. High salinity imposes stress on microbial cells, challenging their survival (Fei Xi et al., 2014; Song et al., 2022). Numerous genes associated with osmoprotective mechanisms, such as salt-in and salt-out mechanisms, membrane modification, protein adaptation, DNA repair, etc. acquired by residents of hypersaline environment for normal growth and development of cells were observed to be prevalent in our study. These genes include ABC-2 type transport system permease protein (K01992), ABC-2 type transport system ATP-binding protein (K01990), ATP-binding cassette, subfamily B, bacterial (K06147), 1-acyl-sn-glycerol-3-phosphate acyltransferase (K00655), 4-hydroxy-tetrahydrodipicolinate synthase (K01714), and DNA helicase II / ATP-dependent DNA helicase PcrA (K03657) (Sleator and Hill, 2002; Yu et al., 2018; Schmitz et al., 2020).

Salt pans in Goa are influenced by three seasons: pre-monsoon, monsoon, and post-monsoon, where salinity ranges from 30–300 PSU (Mani et al., 2012). Therefore, bacteria with different salt tolerance like slightly, moderately, and extremely halophilic bacteria are expected to inhabit this ecosystem. Bacteria within the moderately halophilic group are very important as they occupy a large proportion of halophiles. As adaptive measures, the production of compatible solutes and hydrolytic enzymes to acclimatize to salinity variations is attracting industries (Baati et al., 2010; Ramos-Cormenzana, 2020). For example, the moderate halophilic genus *Micrococcus* synthesizes catalase that plays a role in maintaining colour and flavor in cured meats (Onishi and Kamekura, 1972; García-López et al., 1999). Secondly, *Rhodopirellula,* an abundant genus observed in NC sample is a moderate halophile and was reported to have a role as a food supplement to *Daphina magna* (indicator used for testing toxicity of water) (da Conceição Marinho et al., 2019). Therefore, profound knowledge and discovery of halophilic bacteria in such an ecosystem will help us to disclose numerous properties, enzymes, and proteins of halophiles that could be substantially important to different biotechnological industries.

## Conclusion

Salt pans being polyextremophilic, are dynamic habitats of the resident bacteria potentially capable of tolerating extremely stressful environmental factors, i.e., high salinity (30–450 PSU), high alkalinity (pH 7.6–8.25) and fluctuating temperatures (20°C – 45°C). This is the first study profiling the bacterial communities in the Agarwado, Curca, and Nerul salt pans of Goa. The data reveals that the highest bacterial genera prevail in the Nerul salt pan sediment (250) as compared to Agarwado (201) and Curca (143). However, in the sediments of Agarwado salt pan, 125 distinct genera were found, followed by Nerul (119) and Curca (28) which were exclusively unique to these salt pans. These hypersaline natives are resistant to osmotic stress, demonstrated by the presence of genes in the predicted functional gene profile, that allows them to actively participate in the carbon, nitrogen, and sulfur cycles in these hypersaline environments. A wide microbial community and its function remains unknown, as “unculturable” as it is impractically impossible to mimic these diverse physical, chemical, biological, and mechanical factors which contribute to simulating the salt pan ecosystem in a laboratory. The high throughput sequencing has made it possible to provide an insight of the bacterial community and most of its functions in the salt pans, which could not be explored earlier. Further, a deep understanding of their functional mechanisms in this habitat will help us to assess the existence of these unique bacterial communities, and the adaptive cellular machinery/ biomolecules involved in making this hypersaline environment a conducive environment to thrive in.

## Data availability statement

The datasets presented in this study can be found in online repositories. The names of the repository/repositories and accession number(s) can be found in the article/Supplementary material.

## Author contributions

PG: writing original draft preparation, methodology, and software analysis. SK: supervision, experimental design, review and editing. All authors contributed to the article and approved the submitted version.
