# Supplementary material for: Bacterial diversity and community structure of salt pans from Goa, India
Source: Front Microbiol. 2023 Dec 4;14:1230929. doi: 10.3389/fmicb.2023.1230929 (PMC10726047; doi:10.3389/fmicb.2023.1230929)
Supplement: Supplementary file 1 [file Data_Sheet_1.docx]

Supplementary Material

**Bacterial Diversity and Community Structure of Salt Pans from Goa, India**

Priti Gawas^1^, Savita Kerkar^1*^

^1^School of Biological Sciences and Biotechnology, Goa University, Taleigao Plateau, Goa, 403206, India

*Corresponding author: Dr. Savita Kerkar, Professor and Dean of School of Biological Sciences and Biotechnology, Goa University. E-mail: [drsavitakerkar@gmail.com](mailto:drsavitakerkar@gmail.com), Telephone: 9284693269

**Supplementary data**

**Supplementary Table 1** Common bacterial phyla found in Agarwado salt pan (AC), Curca salt pan (CC), and Nerul salt pan (NC) of Goa, India

| **Samples** | **Common phyla** | **Number of common phyla** |
| --- | --- | --- |
| AC, CC, NC | *Pseudomonadota, Bacillota, Cyanobacteriota* ''*Candidatus* Patescibacteria'', *Bacteroidota, Actinomycetota, Chloroflexota, Acidobacteriota, Planctomycetota,* "*Candidatus* Acetithermota"*, Campylobacterota, Fusobacteriota, Myxococcota, Desulfobacterota, Nitrospirota, Calditrichota, Gemmatimonadota, Verrucomicrobiota, Spirochaetota,* ''*Candidatus* Moduliflexota''*,* ''*Candidatus* Sumerlaeota'', ''*Candidatus* Zixiibacteriota'', *Deinococcota, Fibrobacterota,* ''*Candidatus* Babelota'', ''*Candidatus* Dadaibacteriota'', *Bdellovibrionota,* ''*Candidatus* Hydrogenedentota'', *Elusimicrobiota*Groups at phylum level:WS2, TA06, NB1-j, SAR324 clade (Marine group B) | 33 |
| AC NC | *'Halanaerobiaeota'',* "*Candidatus*Methylomirabilota"  Groups at phylum level: NKB15 | 3 |
| CC NC | *Thermodesulfobacteriota, "Candidatus*Latescibacterota*", Nitrospinota, "Candidatus* Poribacteriota*", Caldisericota, Armatimonadota, "Candidatus*Margulisiibacteriota*", "Candidatus* Neomarinimicrobiota*"*  Groups at phylum level: NKB15, 10bav-F6, MBNT15, LCP-89, WOR-1, Sva0485, CK-2C2-2 | 14 |

**Supplementary Table 2** Common and unique bacterial genera found in Agarwado salt pan (AC), Curca salt pan (CC), and Nerul salt pan (NC) of Goa, India

| **Samples** | **Common genera** | **Number of genera** |
| --- | --- | --- |
| AC, CC, NC | *Robiginitalea, Spirochaeta, Sulfurimonas, Sulfurivermis, Pseudohaliea, Blastopirellula, Fusibacter, Desulfatiglans, Desulfovermiculus, Pelolinea, Desulfobacca, Streptococcus, Marinobacter, Woeseia, Haliangium,* "*Candidatus* Sumerlaea"*, Flavobacterium, Truepera, Nitrospira, Thiohalorhabdus,* "*Candidatus* Omnitrophus"*, Pirellula, Bryobacter, Peredibacter, Bdellovibrio*  Groups at genus level: SM23-31, Subgroup 10, SM1A02, P3OB-42, Pir4 lineage, Subgroup 23, JTB255 marine benthic group | 32 |
| AC, CC | *Sphingobacterium, Sulfurovum, Chryseobacterium,* "*Candidatus* Paceibacter", *Serratia, Enhydrobacter, Pseudomonas, Staphylococcus, Desulfatirhabdium, Delftia,* "*Candidatus* Obscuribacter", *Burkholderia, Tenacibaculum*  Groups at genus level: CL500-3 | 14 |
| AC, NC | *Psychrobacter, Halanaerobium, Antarcticibacterium, Pedobacter, Rhodopirellula, Bacteroides, Bacillus, Planctomicrobium, Acinetobacter, Prevotella, Haemophilus, Guyparkeria, Acanthopleuribacter, Pontibacillus, Tangfeifania, Faecalibacterium, Tumebacillus, Paenibacillus, Simkania, Hydrogenispora, Lachnospira, Halopeptonella, Halanaerobacter, Cerasicoccus, Holdemanella, Desulfovibrio, Salisaeta, Succiniclasticum, Thalassobaculum*  Groups at genus level: BBMC-4 | 30 |
| CC, NC | *Actibacter, Deferrisoma, Propionigenium*, "*Candidatus* Anammoximicrobium", *Calorithrix, Gimesia, Pelagibius*, *Ilumatobacter,* "*Candidatus* Tenderia", *Sandaracinus, Thiohalophilus, Thiogranum, Anderseniella, Magnetovibrio, Roseibacillus,* "*Candidatus* Electrothrix", "*Candidatus* Thiobius", *Desulfosarcina, Limibaculum, Ardenticatena, Nitrospina, Geothermobacter, Inmirania, Fulvivirga, Magnetospira, Mariprofundus, Pseudohongiella, Phaeodactylibacter, Desulfobulbus, Thermomarinilinea, Varunaivibrio, Draconibacterium, Spirochaeta, Imperialibacter, Owenweeksia, Malaciobacter, Nitrosomonas, Luteolibacter, Desulfobacter, Ignavibacterium, Phormidium*, *Halobacteriovorax, Vicingus, Turneriella, Salinirepens, Fluviicola, Thiomicrorhabdus, Phycisphaera, Oceanococcus, Carboxylicivirga, Caldithrix*, ["*Candidatus* Latescibacter"](https://lpsn.dsmz.de/genus/latescibacter), *Oleiphilus, Phaselicystis, Salinibacter, Aureispira, Thermoanaerobaculum*  Groups at genus level: R76-B128, Sva0081 sediment group, Urania-1B-19 marine sediment group, ADurb.Bin120, IS-44, IheB3-7, OM27 clade, A4b, MSBL7, GWE2-31-10, BD1-7 clade, LCP-80 | 69 |
| AC | *Planococcus, Rhodococcus, Pantoea, Exiguobacterium, Sphingomonas, Planomicrobium, Micrococcus, Paracoccus, Microbacterium, Rothia, Massilia, Corynebacterium, Devosia, Pontibacter, Dietzia, Thiohalospira, Mycobacterium, Aerococcus, Microvirga, Methylobacterium, Desulfofustis, Actinomyces, Hyphomicrobium, Methylibium, Roseomonas, Rubrobacter, Pontimonas, Dongia, Chthoniobacter, Porphyromonas, Leptolyngbya,* "*Candidatus* Udaeobacter", *Gordonia, Granulicatella, Gemella, Rubellimicrobium, Nocardioides, Cetobacterium, Chryseolinea, Ferruginibacter, Chryseomicrobium, Rhodocytophaga, Veillonella, Gelidibacter, Abditibacterium, Fusobacterium, Deinococcus, Blastocatella, Tunicatimonas, Nonlabens, Lysobacter, Acidaminococcus, Nibrella, Taibaiella, Desulfocarbo, Ornithinimicrobium, Arcicella, Megamonas, Aridibacter, Cupriavidus, Solimonas, Desulfotignum, Domibacillus, Prosthecobacter, Solirubrobacter, Leptotrichia, Gaetbulibacter, Weeksella, Flavitalea, Lysinibacillus, Rufibacter, Panacagrimonas, Herbaspirillum, Glutamicibacter, Enterococcus, Brevibacillus, Brevibacterium, Dyadobacter, Virgisporangium, Bergeyella, Anaerolinea, Ottowia, Treponema, Hymenobacter, Verticiella, Monoglobus, Carnobacterium, Halothiobacillus, Natranaerobius, Alloprevotella, Desulfitobacterium,* "*Candidatus* Protochlamydia", *Wenzhouxiangella, Prosthecochloris, Pseudonocardia, Arcticibacter,* "*Candidatus* Halysiosphaera", *Propioniciclava, Singulisphaera, Ohtaekwangia, Ahniella, Jeotgalicoccus, Alkalibacterium, Halarsenatibacter, Nubsella, Neochlamydia, Pajaroellobacter, Streptomyces, Anaerococcus, Gaiella, Cutibacterium, Ezakiella, Chloronema, Mycoplasma*  Groups at genus level: TM7, RS25G, Ellin516, AKYG587, SH-PL14, Ellin6067, SWB02, SC103, MND1, RB41, *Christensenellaceae* R-7 group | 125 |
| CC | *Aeromonas, Klebsiella, Stenotrophomonas,* *Bradyrhizobium, Ralstonia, Allorhizobium, Endomicrobium, Porticoccus, Litoribrevibacter, Sulfuriflexus, Hyphococcus, Roseivirga, Methylophaga, Hydrogenovibrio, Litorivivens, Oleibacter, Desulfomicrobium, Saccharopolyspora, Gracilimonas,* "*Candidatus* Actinomarina", *Desulfurivibrio, Chlamydia, Alterococcus,* "*Candidatus* Kuenenia"  Groups at genus level: BCf9-17 termite group, ADurb.Bin063-1, SUP05 cluster, MSBL3 | 28 |
| NC | *Arthrospira, Defluviicoccus, Clostridiisalibacter, Thiohalomonas, Oceanirhabdus, Methyloceanibacter, Silicimonas, Roseivivax, Sporacetigenium, Bradymonas, Orenia, Roseicyclus, Iodidimonas, Limibacillus, Rubripirellula, Oscillatoria, Parahaliea, Pullulanibacillus, Haloferula, Pelagicoccus, Desulfosalsimonas, Cyanobacterium, Sporosalibacterium, Natranaerovirga, Tepidibacillus, Lentimonas,* "*Candidatus* Thiodiazotropha", *Crocinitomix, Ruminiclostridium, Desulfomonile, Ferrimonas, Desulfofaba, Limisphaera, Vulcanibacillus, Marinobacterium, Halobacillus, Anaerophaga, Haloplasma, Balneola, Magnetococcus, Gottschalkia, Dichotomicrobium, Kordiimonas, Halofilum, Geoalkalibacter, Alicyclobacillus, Microbulbifer, Maricaulis, Rhodovibrio, Synechocystis, Asteroleplasma, Izimaplasma, Maritimimonas, Succinivibrio, Clostridium, Thermoflexus, Cyanothece*, *Bythopirellula*, *Waddlia*, *Oceanicaulis, Aliifodinibius*, *Pleurocapsa*, *Halodesulfovibrio*, *Roseimaritima*, *Brachyspira*, *Filomicrobium*, *Puniceicoccus*, *Sunxiuqinia*, *Anaeromicrobium*, *Alcanivorax*, *Geminocystis*, *Coxiella*, *Sedimenticola*, *Bifidobacterium, Geitlerinema*, *Fodinibius, Winogradskyella,* "*Candidatus* Endecteinascidia", *Levilinea, Dehalobacterium, Desulfospira, Muricauda, Parabacteroides, Vibrio, Microscilla, Lewinella, Marinilabilia, Escherichia, Halanaerobaculum, Desulfitibacter, Dialister, Alistipes, Megasphaera, Marinicella, Pseudobacteriovorax, Halomonas, Aquicella, Rubidibacter*, *Amphiplicatus, Anaerovibrio, Schleiferia,* "*Candidatus* Nucleicultrix", *Heliophilum*  Groups at genus level: SBZC-1223, YC-ZSS-LKJ90, Cm1-21, RBG-16-49-21, SCGC AAA164-E04, RCP1-48, M2PT2-76 termite group, YC-ZSS-LKJ63, UCG-002, Sva0996 marine group, B2706-C7, UCG-012, BMS9AB35, C1-B045, JL-ETNP-F27, MAT-CR-P4-C12 | 119 |
