# Supplementary material for: Bacterial diversity and community structure of salt pans from Goa, India
Source: Front Microbiol. 2023 Dec 4;14:1230929. doi: 10.3389/fmicb.2023.1230929 (PMC10726047; doi:10.3389/fmicb.2023.1230929)

Supplementary Material

**Bacterial Diversity and Community Structure of Salt Pans from Goa, India**

Priti Gawas^1^, Savita Kerkar^1*^

^1^School of Biological Sciences and Biotechnology, Goa University, Taleigao Plateau, Goa, 403206, India

*Corresponding author: Dr. Savita Kerkar, Professor and Dean of School of Biological Sciences and Biotechnology, Goa University. E-mail: [drsavitakerkar@gmail.com](mailto:drsavitakerkar@gmail.com), Telephone: 9284693269

**Supplementary data**

**Supplementary Figure 1** Heatmap showing genes associated with carbon metabolism predicted by using PICRUST2 algorithm in the sediment samples of Agarwado salt pan (AC), Curca salt pan (CC), and Nerul salt pan (NC) of Goa, India**.**


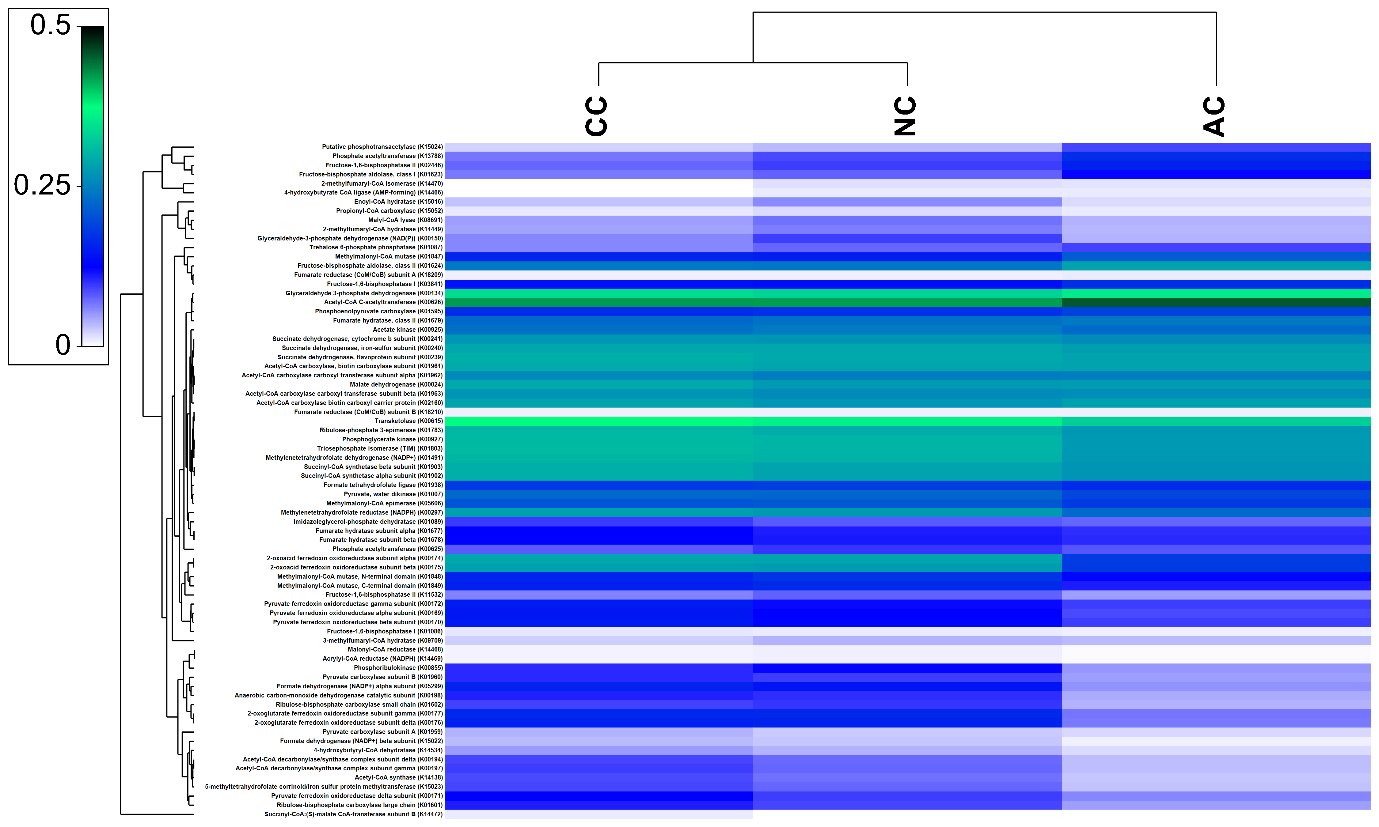


**Supplementary Figure 2** Heatmap showing genes associated with nitrogen metabolism predicted by using PICRUST2 algorithm in the sediment samples of Agarwado salt pan (AC), Curca salt pan (CC), and Nerul salt pan (NC) of Goa, India.


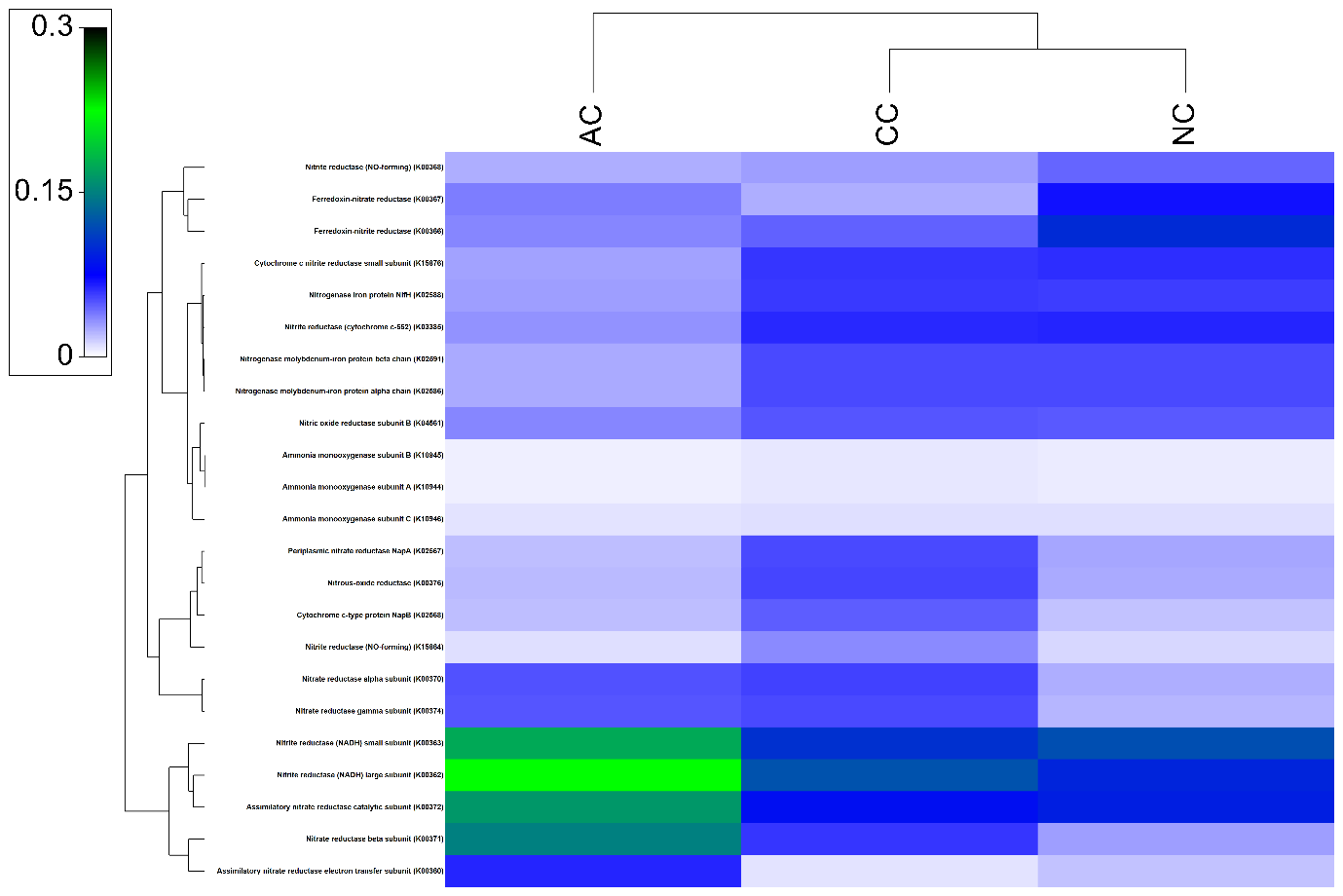


**Supplementary Figure 3** Heatmap showing genes associated with sulfur metabolism predicted by using PICRUST2 algorithm in the Agarwado salt pan (AC), Curca salt pan (CC), and Nerul salt pan (NC) of Goa, India.


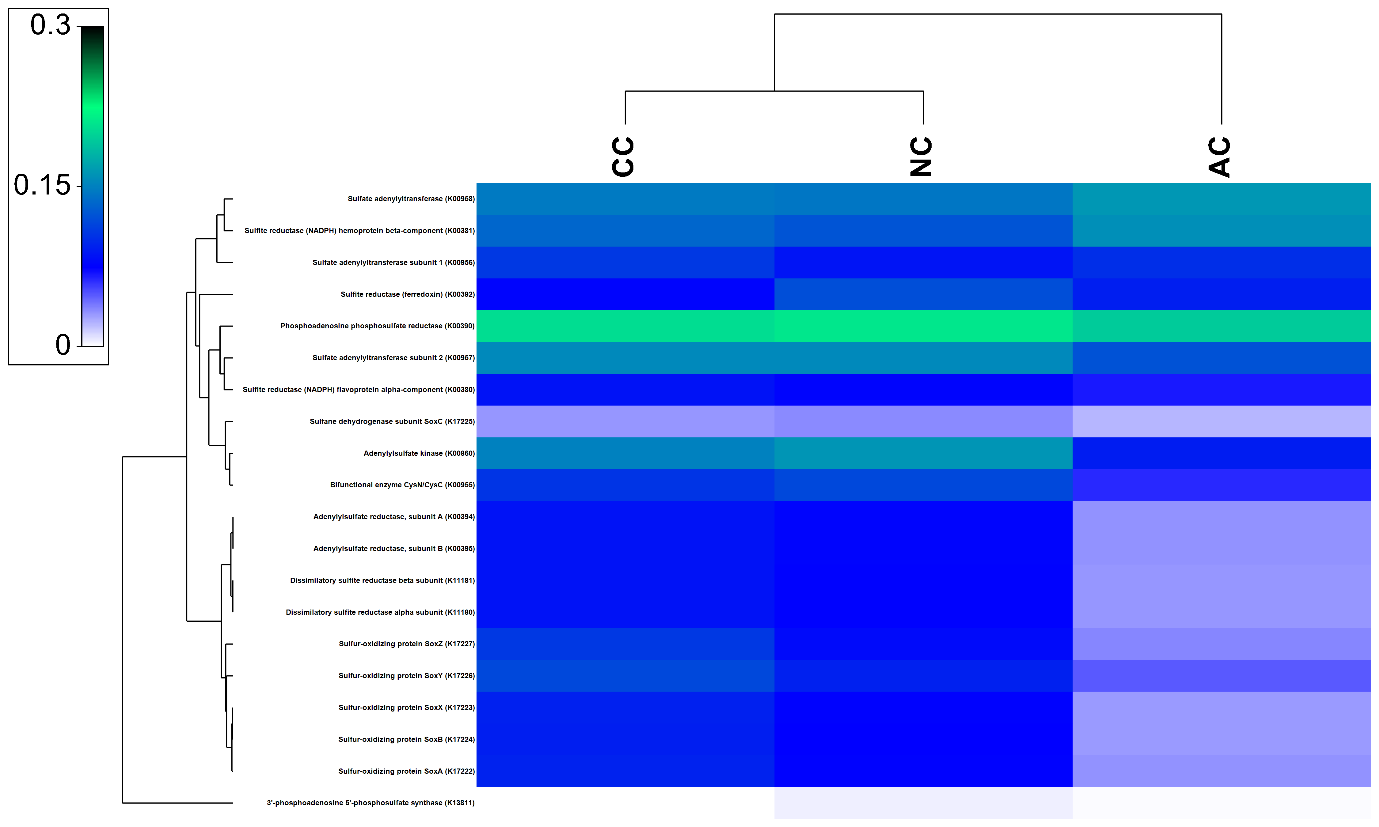

Supplement: Supplementary file 2 [file Data_Sheet_2.docx]
